# Pulse pressure and the risk of renal hyperfiltration in young adults: Results from Korea National Health and Nutrition Examination Survey (2010–2019)

**DOI:** 10.3389/fmed.2022.911267

**Published:** 2022-09-13

**Authors:** Eunji Yang, Sang Ho Park, Seoyoung Lee, Donghwan Oh, Hoon Young Choi, Hyeong Cheon Park, Jong Hyun Jhee

**Affiliations:** ^1^Division of Nephrology, Department of Internal Medicine, Gangnam Severance Hospital, Yonsei University College of Medicine, Seoul, South Korea; ^2^Department of Internal Medicine, Gangnam Severance Hospital, Yonsei University College of Medicine, Seoul, South Korea; ^3^Severance Institute for Vascular and Metabolic Research, Yonsei University College of Medicine, Seoul, South Korea

**Keywords:** pulse pressure, renal hyperfiltration, estimated glomerular filteration rate, young adult, kidney function

## Abstract

**Background:**

High pulse pressure (PP) is associated with increased risk of decline of kidney function. However, little is known about the association between PP and RHF in young adults. This study aimed to evaluate the association between PP and RHF in healthy young adults.

**Methods:**

Data were retrieved from the Korea National Health and Nutrition Examination Survey from 2010 to 2019. A total of 10,365 participants aged 19–39 years with no hypertension and normal kidney function were analyzed. RHF was defined as logarithm transformed estimated glomerular filtration rate (eGFR) with residuals >90th percentile after adjustment for sex, logarithm transformed age, weight, and height. Participants were divided into tertile based on PP levels.

**Results:**

The prevalence of RHF was higher in higher PP tertile group (6.6, 10.5, and 12.7% in T1, T2, and T3; *P* for trend < 0.001). In multivariable logistic regression analyses, the risk for RHF was increased in higher PP tertiles compared to the lowest tertile [odds ratio (OR), 1.42; 95% confidence interval (CI), 1.19–1.69 in T2; OR, 1.44; 95% CI, 1.20–1.73 in T3]. When PP levels were treated as continuous variable, the risk of RHF was increased 2.36 per 1.0 increase of PP (*P* < 0.001). In subgroup analyses stratified sex, histories of diabetes or dyslipidemia, and isolated systolic hypertension or isolated diastolic hypertension, there were no significant interactions with PP for the risk for RHF, suggesting that high PP was associated with increased risk of RHF regardless of subgroups. However, the subgroup with BMI showed significant interaction with PP for the risk of RHF, indicating that participants with BMI ≥ 25 kg/m^2^ were at higher risk of RHF with increasing PP levels than those with BMI < 25 kg/m^2^ (OR, 1.89; 95% CI, 1.25–2.87 in BMI < 25 kg/m^2^; OR, 3.16; 95% CI, 1.74–5.73 in BMI ≥ 25 kg/m^2^; *P* for interaction = 0.01).

**Conclusion:**

High PP is associated with an increased risk of RHF in healthy young adults and this association is prominent in obese young adults. The assessment of PP and associated RHF may give benefit to early detect the potential risk of CKD development in young adults.

## Introduction

Chronic kidney disease (CKD) is one of the representative health problems and its incidence is increasing worldwide (1, 2). Once kidney failure begins, it is difficult to recover, eventually leads to end-stage kidney disease (ESKD) requiring dialysis or transplantation. Moreover, patients with CKD have an increased risk of cardiovascular complications and death (1, 3). Therefore, early identification and management of risk factors for CKD is crucial. Meanwhile, in recent years, health problems such as hypertension, diabetes, and obesity initiating from young adults are attracting attention (4). Despite the fact that the onset of a health problem at younger age is associated with an increased risk of adverse clinical outcomes, the proportion of young adults who receive medical care is relatively low (5, 6). Given the progressive nature of kidney disease, early identification of the risk factors for CKD in young adults may prevent the development of CKD and reduce the burden of medical expenses.

Renal hyperfiltration (RHF), an abnormally increased status of glomerular filtration rate (GFR), is one of the known risk factors for kidney function deterioration (7–9). In addition, RHF is viewed as a marker of vascular dysfunction and increased arterial stiffness. Physiologically, high protein intake or pregnancy or obesity increase GFR (10–12). High intra-glomerular pressure and shear stress may damage the nephron and lead to detachment of glomerular epithelial cells from the glomerular capillary wall, contributing to the progression of kidney injury (13, 14). Thus, abnormally increased GFR serves as an early sign of kidney disease (3).

Pulse pressure (PP) is derived from the difference between systolic blood pressure (SBP) and diastolic BP (DBP) and reflects the hemodynamic changes of BP and flow. The levels of PP are determined by stroke volume, aortic stiffness, and wave reflection and elevated PP levels represents increased arterial stiffness. Reboldi et al. (14) observed that subjects with RHF were associated with an elevated 24-h PP levels and increased risk of cardiovascular events (CVEs). This study suggested that endothelial dysfunction or alteration in microvascular and macrovascular structures are associated with an increased arterial stiffness, which contributes to occurrence of RHF and adverse cardiovascular outcomes.

Considering the increasing rate of health problems in young adults and the progressive nature of kidney disease, it is crucial to identify risk factors for CKD before the overt disease onset. Thus, we aimed to investigate the predictive role of PP, which is an indicator of increased arterial stiffness, on early detection of RHF among young adults.

## Materials and methods

### Study subjects

**FIGURE 1:**
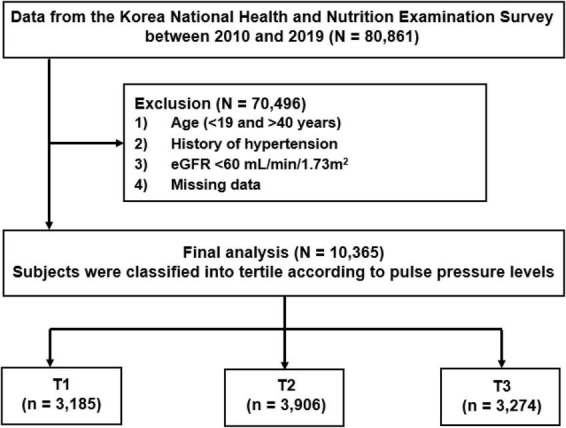
Study T1-3 represents tertile according to pulse pressure levels. T1 is the lowest and T3 is the highest pulse pressure. eGFR, estimated glomerular filtration rate.

Data were retrieved from the Korea National Health and Nutrition Examination Survey (KNHANES 2010–2019). KNHANES is a widespread surveillance system that survey the health and nutrition status of Koreans. Detailed data resource profile about KNHANES was previously reported elsewhere (15). Briefly, KNHANES consists of individual’s health-related performance, quality of life, healthcare utilization, anthropometric measures, biochemical and clinical profiles. It is composed of three component surveys: a health interview, health examination and nutrition survey. The surveys collect detailed information on socioeconomic status, health behaviors, quality of life, healthcare utilization, anthropometric measures, biochemical profiles using fasting blood serum and urine, measures for dental health, vision, hearing and bone density, X-ray test results food intake and dietary behavior (15, 16). For this study, a total of 80,861 participants were screened. The participants were excluded from the study with an age of under 19 or over 40 years, past history of hypertension, eGFR less than 60 ml/min/1.73 m^2^, and missing data. Finally, 10,365 participants aged 19–39 years with normal kidney function and without hypertension were included for the study analysis. The participants were divided into tertile based on PP levels [the lowest tertile of PP as T1 (*n* = 3,185), middle tertile of PP as T2 (*n* = 3,906), and the highest tertile as T3 (*n* = 3,274)] (Figure 1). All subjects voluntarily participated in the study and provided informed consent. This study was performed in accordance with the Declaration of Helsinki and approved by the Institutional Review Board (IRB) of the Centers for Disease Control and Prevention in Korea (KNHANES 2010-2014 IRB approvals; 2010-02CON-21-C, 2011-02CON-06-C, 2012-01EXP-01-2C, 2013-07CON-03-4C, and 2013-12EXP-03-5C, IRB approval was not required for KNHANES 2015-2017 because this survey was conducted for the purpose of public welfare, KNHANES 2018-2019 IRB approvals; 2018-01-03-P-A and 2018-01-03-C-A).

### Data collection

Demographic and socioeconomic data, including age, sex, levels of education and income, smoking status, alcohol intake, and medical histories. Education status was classified as low level for those who graduated from elementary school to middle school and as high level for those who graduated from high school to graduated university. Households with an average monthly income of less than 2 million won were classified as low income level, and those with more than 2 million won were classified as high income level. For the past medical histories, diabetes, dyslipidemia, and cardiovascular disease (CVD) were defined as a medical diagnosis. Anthropometric indices including height, weight, SBP, DBP were examined by skilled study workers. BPs were measured by special investigation nurse in the Korea Centers for Disease Control and Prevention. Participants sat in a comfortable position after they had rested for at least 5 min and had not smoked within 30 min of the measurements. BP was measured on three consecutive occasions in a relaxed environment. The mean of the second and the third measurements was adopted for the data analysis (17). PP was calculated by the difference between SBP and DBP. Blood and urine samples were analyzed for creatinine, hemoglobin, fasting plasma glucose, HbA1c, Total cholesterol, and low-density lipoprotein-cholesterol (LDL-C). Proteinuria was measured by the dipstick urine analysis method using Urisys 2400 in 2010–2018 (Roche, Germany) and UC-3500 in 2019 (Sysmex/Japan). The dipstick urine test results were described as negative, trace, 1+, 2+, 3+, or 4+. Proteinuria was defined as more than trace levels. Serum creatinine level was measured by the Jaffe rate-blanked and compensated method using Hitachi Automatic Analyzer 7600-210 in 2019–2018 (Hitache, Japan) and by the Kinetic colorimetric assay method using Cobas in 2019 (Roche, Germany). The estimated glomerular filtration rate (eGFR) was calculated by the CKD-Epidemiology Collaboration (CKD-EPI) equation (18).

### Definition of renal hyperfiltration

The study endpoint was RHF which was defined by using the previously reported method with modification (19). In detail, residuals were calculated from linear regression analysis with logarithm transformed eGFR as dependent variable and logarithm-transformed age, sex, weight, and height as independent variables. RHF was defined with residuals of >90th percentile from the model.

### Statistical analysis

All statistical analyses were performed using IBM SPSS software for Windows version 25.0 (IBM Corporation, Chicago, IL, United States). Continuous variables were expressed as mean ± standard deviation and categorical variables as absolute numbers with percentages. All data were tested for normality before the statistical analysis. Baseline characteristics of tertiles classified by PP levels were compared by using analysis of variance test for continuous variables, and the chi-squared test or Fisher’s exact test for categorical variables. Pearson’s correlation analysis was performed to identify associated factors with PP levels. To evaluate the association between PP and RHF, multivariable logistic regression analysis was performed with adjustment for covariates such as age, sex, body mass index (BMI), income and education status, alcohol and smoking status, history of diabetes, hemoglobin, total cholesterol, and proteinuria. Additionally, Receiver operating characteristic (ROC) curve analysis was performed to determine the cutoff values of PP and corresponding SBP and DBP levels for the risk of RHF. Subgroup analysis was performed stratified by sex, BMI (<25 or ≥25 kg/m^2^), histories of diabetes or dyslipidemia, and isolated systolic hypertension (ISH) or isolated diastolic hypertension (IDH). Based on the 2017 ACC/AHA criteria, ISH was defined as SBP ≥ 130mmHg and DBP < 80mmHg and IDH was defined as SBP < 130mmHg and DBP ≥ 80mmHg (20). *P* for interactions for the risk of RHF were assessed between each subgroup and PP levels. Two-sided *P* value < 0.05 were considered statistically significant.

## Results

### Baseline characteristics of the study subjects

A total of 10,365 participants were analyzed for this study (Figure 1). The baseline characteristics according to tertile of PP are shown in Table 1. The mean age of study participants were 30.1 ± 6.1 years and 5,716 (55.1%) were female. The mean levels of eGFR in study participants were 110.3 ± 23.6 ml/min/1.73m^2^ and that of PP were 36.6 ± 7.5mmHg. The mean levels of PP were 28.6 ± 3.1, 35.9 ± 2.0, 45.2 ± 5.4 mmHg in T1, T2, and T3, respectively. Participants in higher tertiles were more likely to be young and male. Participants in higher tertiles had higher levels of BMI, were more frequent smoker, had lower education status and less income, had higher SBP and lower DBP, had more frequent past histories of dyslipidemia or CVDs compared to the lowest tertile. In laboratory tests, participants in higher tertiles showed higher levels of eGFR, hemoglobin, fasting plasma glucose, HbA1c and lower levels of total cholesterol and LDL-C than the lowest tertile. As previous studies reported that PP levels are higher in male than female in young adults, we further evaluated the differences in baseline characteristics according to the PP levels between male and female (Supplementary Tables 1, 2) (21, 22). The mean levels of PP were higher in male compared with female in this study (38.6 ± 8.0 mmHg in male and 35.0 ± 6.7 mmHg in female, *P* < 0.001). The male participants in higher PP tertiles were more frequent smoker. However, female participants did not show differences in smoking status according to tertiles of PP. In laboratory test, male participants with higher tertiles of PP showed no difference in hemoglobin levels, whereas female participants with higher tertile showed low levels of hemoglobin. In addition, male participants with higher tertile showed lower levels of total cholesterol, whereas female did not.

**TABLE 1:** Baseline characteristics.

	Pulse pressure			
	
Characteristics	T1 (*n* = 3,185)	T2 (*n* = 3,906)	T3 (*n* = 3,274)	*P*
**Demographic data**				
Age, years	31.0 ± 5.9	30.5 ± 6.0	28.8 ± 6.3	<0.001
Sex				<0.001
Male, n (%)	1018 (32.0%)	1700 (43.5%)	1931 (59.0%)	
Female, n (%)	2167 (68.0%)	2206 (56.5%)	1343 (41.0%)	
BMI, kg/m^2^	22.3 ± 3.5	23.0 ± 3.7	24.0 ± 4.1	<0.001
Smoking status, n (%)	1057 (33.2%)	1458 (37.3%)	1435 (43.8%)	<0.001
Alcohol status, n (%)	3094 (30.9%)	3778 (37.7%)	3153 (31.5%)	0.071
SBP, mmHg	101.9 ± 7.4	108.1 ± 8.2	116.0 ± 9.4	<0.001
DBP, mmHg	73.3 ± 7.1	72.2 ± 8.0	70.8 ± 8.8	<0.001
Education, n (%)				<0.001
Low	57 (1.8%)	84 (2.2%)	75 (2.3%)	
High	3128 (98.2%)	3822 (97.9%)	3199 (97.7%)	
Income, n (%)				0.002
Low	1511 (47.4%)	1912 (49.0%)	1666 (50.9%)	
High	1674 (52.6%)	1994 (51.0%)	1608 (49.1%)	
**Comorbidities, n (%)**				
Diabetes	24 (0.8%)	16 (0.4%)	26 (0.8%)	0.076
Dyslipidemia	58 (1.8%)	87 (2.2%)	62 (1.9%)	0.014
Cardiovascular disease	2 (0.06%)	1 (0.02%)	2 (0.06%)	0.034
**Laboratory data**				
eGFR, ml/min/1.73 m^2^	109.4 ± 12.5	110.3 ± 12.6	111.2 ± 12.6	<0.001
Proteinuria, n (%)	482 (15.1%)	497 (12.7%)	499 (15.2%)	0.006
Hemoglobin, g/dl	14.0 ± 1.5	14.2 ± 1.6	14.5 ± 1.7	<0.001
Fasting plasma glucose, mg/dL	90.8 ± 12.4	91.7 ± 16.5	92.3 ± 13.5	<0.001
HbA1c, %	5.37 ± 0.5	5.38 ± 0.5	5.40 ± 0.5	0.141
Total cholesterol, mg/dl	185.0 ± 34.0	184.0 ± 32.7	182.4 ± 32.6	0.005
LDL-C, mg/dl	108.7 ± 29.9	107.7 ± 29.7	105.6 ± 29.7	<0.001

Data are presented as mean ± SD and number (%).

BMI, body mass index; SBP, systolic blood pressure; DBP, diastolic blood pressure; eGFR, estimated glomerular filtration rate; LDL-C, low density lipoprotein-cholesterol; SD, standard deviation.

### Factors associated with the pulse pressure levels

Next, we performed Pearson‘s correlation analysis to evaluate the modifiable factors which are associated with high PP levels (Table 2). Male sex (*r* = 0.24, *P* < 0.001), BMI (*r* = 0.19, *P* < 0.001), smoking (*r* = 0.09, *P* < 0.001), SBP (*r* = 0.61, *P* < 0.001), eGFR (*r* = 0.07, *P* < 0.001), hemoglobin (*r* = 0.15, *P* < 0.001), and fasting plasma glucose (*r* = 0.04, *P* < 0.001) levels were positively associated with PP levels. In contrast, age (*r* = –0.17, *P* < 0.001), DBP (*r* = –0.17, *P* < 0.001) and total cholesterol (*r* = –0.04, *P* < 0.001) levels were negatively associated with PP levels.

**TABLE 2:** The factors associated with pulse pressure.

	PP	Age	Sex*	BMI	Smoke*	SBP	DBP	eGFR	Hb	FPG	TC
PP r *P*	1 –	–0.17 <0.001	0.24 <0.001	0.19 <0.001	0.09 <0.001	0.61 <0.001	–0.17 <0.001	0.07 <0.001	0.15 <0.001	0.04 <0.001	–0.04 <0.001
Age r *P*		1 –	–0.06 <0.001	0.10 <0.001	0.08 <0.001	–0.02 0.083	0.14 <0.001	–0.36 <0.001	–0.11 <0.001	0.16 <0.001	0.21 <0.001
Sex r *P*			1 –	0.29 <0.001	0.47 <0.001	0.44 <0.001	0.32 <0.001	–0.28 <0.001	0.78 <0.001	0.10 <0.001	0.07 <0.001
BMI r *P*				1 –	0.18 <0.001	0.38 <0.001	0.30 <0.001	–0.11 <0.001	0.27 <0.001	0.25 <0.001	0.24 <0.001
Smoke r *P*					1 –	0.22 <0.001	0.20 <0.001	–0.16 <0.001	0.40 <0.001	0.10 <0.001	0.09 <0.001
SBP r *P*						1 –	0.68 <0.001	–0.10 <0.001	0.39 <0.001	0.16 <0.001	0.12 <0.001
DBP r *P*							1 –	–0.20 <0.001	0.35 <0.001	0.16 <0.001	0.19 <0.001
eGFR r *P*								1 –	–0.26 <0.001	–0.05 <0.001	–0.13 <0.001
Hb r *P* FPG r *P*									1 –	0.12 <0.001 1 –	0.13 <0.001 0.14 <0.001
TC r *P*											1 –

*Sex: male vs.

female; Smoke: yes vs. no. BMI, body mass index; Smoke, history of smoking; SBP, systolic blood pressure; DBP, diastolic blood pressure; eGFR, estimated glomerular filtration rate; Hb, hemoglobin; FPG, fasting plasma glucose; TC, total cholesterol.

### Risk of renal hyperfiltration according to pulse pressure

The prevalence of RHF was significantly higher in higher tertiles (6.6, 10.5, and 12.7% in T1, T2, and T3, respectively; *P* for trend < 0.001). To examine the association between PP and the risk for RHF, logistic regression analyses were performed. In unadjusted model, higher tertiles showed increased risks for RHF compared to lowest tertile [odds ratio (OR), 1.65; 95% confidence interval (CI), 1.39–1.96; *P* < 0.001 in T2; OR, 2.05; 95% CI, 1.73–2.44; *P* < 0.001 in T3). After adjustment for clinical variables including age, sex, BMI, education and income status, alcohol and smoking status, history of diabetes, levels of hemoglobin, total cholesterol, and proteinuria, the increased risks of RHF were observed in higher tertiles compared to the lowest tertile (OR, 1.42; 95% CI 1.19–1.69; *P* < 0.001 in T2; OR, 1.44; 95% CI, 1.20–1.73; *P* < 0.001 in T3). These associations were consistently observed when the PP levels were treated as continuous variable that increasing PP levels (per 1.0 increase of log-transformed values) were associated with 2.36-folded increased risk of RHF in multivariable logistic regression model (OR, 2.36; 95% CI, 1.67–3.32; *P* < 0.001) (Table 3 and Supplementary Table 3).

**TABLE 3:** Risk of RHF according to pulse pressure.

		Model 1		Model 2		Model 3	
				
	Prevalence of RHF, n (%)	OR (95% CI)	*P*	OR (95% CI)	*P*	OR (95% CI)	*P*
Pulse pressure*							
per 1.0 increase		4.98 (3.61–6.86)	<0.001	2.77 (1.98–3.86)	<0.001	2.36 (1.67–3.32)	<0.001
T1	211 (6.6%)	(Reference)					
T2	409 (10.5%)	1.65 (1.39–1.96)	<0.001	1.45 (1.22–1.73)	<0.001	1.42 (1.19–1.69)	<0.001
T3	416 (12.7%)	2.05 (1.73–2.44)	<0.001	1.55 (1.30–1.86)	<0.001	1.44 (1.20–1.73)	<0.001

*Log-transformed.

Model 1: Unadjusted model.

Model 2: Adjusted for age, sex.

Model 3: Adjusted for age, sex, BMI, income, education, alcohol and smoking status, history of diabetes, hemoglobin, total cholesterol, proteinuria.

BMI, body mass index; OR, odds ratio; CI, confidence interval.

**FIGURE 2:**
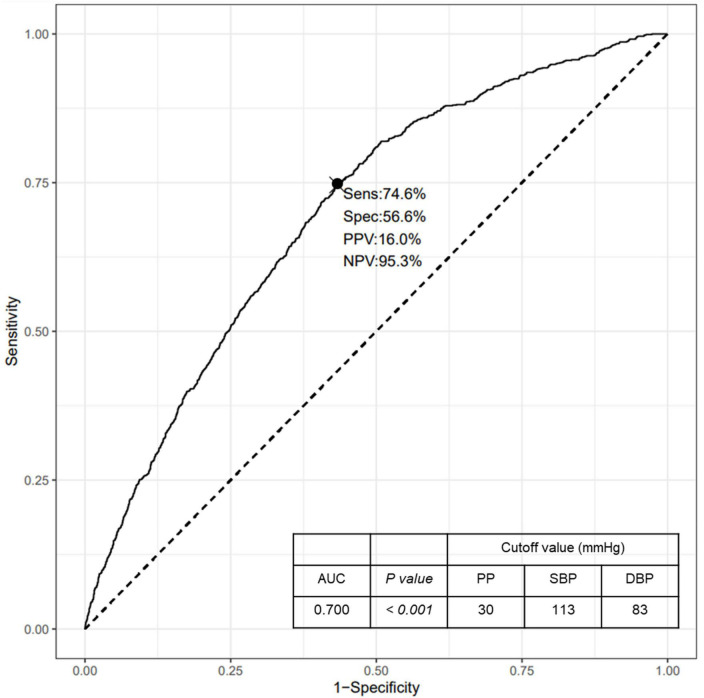
ROC curve for the risk of RHF according to the PP levels. ROC, Receiver operating characteristic; RHF, renal hyperfiltration; PP, pulse pressure; Sens, sensitivity; Spec, specificity; PPV, positive predictive value; NPV, negative predictive value; AUC, area under the curve; SBP, systolic blood pressure; DBP, diastolic blood pressure.

We additionally performed ROC curve analysis to determine the cutoff values of PP and corresponding SBP and DBP levels for the risk of RHF (Figure 2). The area under the ROC curve was 0.700 (95% CI, 0.684–0.716, *P* < 0.001) and the sensitivity and specificity of the curve were 74.6 and 56.6%, respectively. The cutoff value of PP levels, which increases the risk of RHF, was 30 mmHg, and the corresponding SBP and DBP levels were 113 and 83 mmHg, respectively.

### Subgroup analyses stratified by sex, BMI, histories of diabetes or dyslipidemia, and ISH or IDH

**FIGURE 3:**
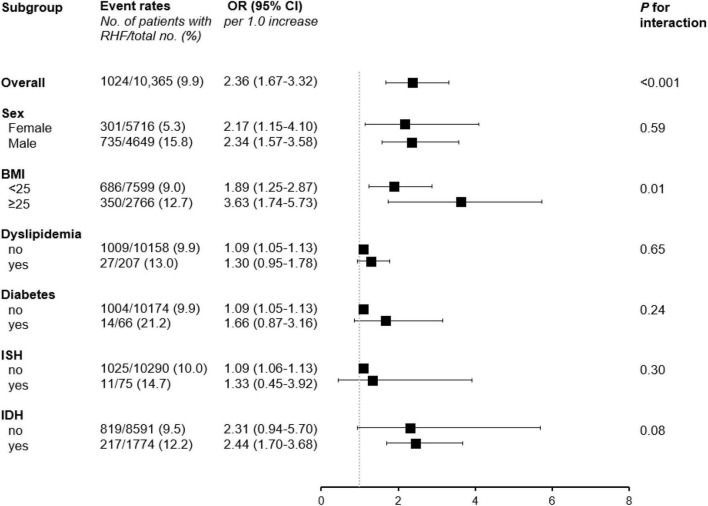
Subgroup analyses stratified by sex, BMI, histories of diabetes or dyslipidemia, and ISH or IDH. ORs were calculated per 1.0 increase of log-transformed PP levels after adjustment for age, sex, BMI, income, education alcohol and smoking status, history of diabetes, hemoglobin, total cholesterol, and proteinuria. RHF, renal hyperfiltration; OR, odds ratio; CI, confidence interval; BMI, body mass index; ISH, isolated systolic hypertension; IDH, isolated diastolic hypertension.

To further confirm the association between PP levels and the risk of RHF, subgroup analyses stratified with sex (female vs. male), BMI (<25 vs. ≥25kg/m^2^), histories of diabetes or dyslipidemia (no vs. yes), and ISH or IDH (no vs. yes) were performed (Figure 3 and Supplementary Table 4). There were no significant interactions between subgroups including sex, histories of diabetes or dyslipidemia, and ISH or IDH and the PP levels for the risk of RHF, suggesting that the association of increased risk for RHF with high PP levels was regardless of sex, histories of diabetes or dyslipidemia, and ISH or IDH. However, the subgroup with BMI showed significant interaction with the PP levels for the risk of RHF, indicating that participants with BMI higher than 25 kg/m^2^ showed an increased risk of RHF with increasing PP levels than those with BMI lower than 25 kg/m^2^ [OR (per 1.0 increase in log-transformed PP), 1.89; 95% CI, 1.25–2.87 in BMI < 25 kg/m^2^; OR (per 1.0 increase in log-transformed PP), 3.16; 95% CI, 1.74–5.73 in BMI ≥ 25 kg/m^2;^ *P* for interaction = 0.01).

## Discussion

The results of the present study using large cohort data of young Korean adults showed that elevated PP was associated with an increased risk of RHF in young adults with normal kidney function. In particular, significant interaction between obesity and PP levels for the risk of RHF was found that obese young adults with BMI larger than 25 kg/m^2^ were more prone to the risk of RHF than those with BMI less than 25 kg/m^2^. These findings suggest that the identification of elevated PP levels in young adults may play a role in early identification of RHF, one of the risk factors for future deterioration of kidney function.

RHF is known to precede progressive kidney injury. RHF refers to the state in which GFR is abnormally increased in the kidney, which cause chronic decline of kidney function (23). Considering the progressive nature of kidney disease, early identification and management of risk factors are crucial to prevent further progression to ESKD needing chronic dialysis or kidney transplantation (24–26). Previous studies have demonstrated that RHF is associated with hypertension, diabetes, obesity, and smoking (27, 28). However, the association between elevated PP levels and the RHF risk is poorly elucidated. In this study, elevated PP levels were associated with an increased risk for RHF. PP, calculated by the difference between SBP and DBP, serves as an indicator of hemodynamic changes in BP and indicates vascular dysfunction including RHF (29, 30). Mechanistically, PP is caused by the close interaction of blood ejected from left ventricle with aorta or large arteries (29). When the elasticity of the aorta or large arteries decreases, the stiffness of those arteries conversely increases with increasing PP levels (31). The increase in PP contributes to vascular endothelial damage, which leads to mechanical fatigue and deepens vascular stiffness. This process viciously worsens central wave reflection and increases the PP levels again (32, 33). The increased PP with increased arterial stiffness derived from the vicious cycle affect the renal vasculature, consequently impairing renal hemodynamics (33). Initially, elevated PP results in glomerular hyperfiltration. However, chronic and steady increase in pressure and arterial stiffness eventually leads to ischemic and fibrotic changes in glomerulus. These changes further lead to loss of renal autoregulation with glomerular hypertrophy and sclerosis, ultimately contributing to nephrosclerosis and decline of kidney function (7, 34).

In this study, participants with high PP levels showed the increased risk of RHF as well as higher proportion of obesity and smoking status, which are known risk factors for CKD. Furthermore, when Pearson’s correlation analysis was performed to evaluate the modifiable factors with high PP levels, male sex, BMI, smoking, and fasting plasma glucose levels showed the positive association with PP levels, whereas age and total cholesterol levels showed the negative association with PP levels. As noted in previous reports, obesity, smoking, and hyperglycemia are associated with elevated PP levels and serve as risk factors for arterial stiffness in young adults similar to older generation (35–39). Thus, modifying these factors might give benefit to overcome deleterious effects of high PP levels in young adults. Meanwhile, younger age and male sex are known to associate with high PP levels in the younger population. Younger men are commonly associated with spurious systolic hypertension or hyperkinetic status which contribute to high PP levels (21, 40). In addition, unlike elderly patients, elevated cholesterol levels without overt atheroma are associated with reduced arterial stiffness in the younger population (41–43). Clear mechanism between these factors and elevated PP levels is unclear and needs to be elucidated in young adults. Nevertheless, early identification of modifiable factors associated with high PP levels, such as obesity, smoking, or hyperglycemia, may have clinical implications for preventing RHF and future kidney dysfunction.

Another interesting finding of the present study is that obese young adults with BMI larger than 25 kg/m^2^ showed more increased risk of RHF compared to those with BMI less than 25 kg/m^2^. RHF is one of the well-known major alterations resulting from obesity-related glomerulopathy (44). The glomerulus enlarges in response to obesity-related changes including RHF and increased renal plasma flow. Although most obese patients have stable or slowly progressive proteinuria, up to one-third develop nephrosclerosis and progressive kidney failure or ESKD (45). Furthermore, obesity is also associated with arterial stiffness or elevated PP even in child to young adults similar to older generation (46, 47). Taken together, even in healthy young adults with no history of hypertension and without vascular damage, concurrent obesity and elevated PP may further accelerate RHF and lead to more rapid and severe progression of renal hemodynamic changes. Resultantly, obese young adults with high PP may face irreversible nephrosclerotic changes, kidney failure, and initiation of dialysis at an early age. Therefore, the findings of this study suggest that young adults, particularly those with obesity, need monitoring of PP levels and associated RHF to prevent future development of kidney disease.

Given that high PP levels or ISH are typical in male than female in young adults, there may exist sex-disparity in the association between high PP levels and RHF. In this study, the mean levels of PP were higher in male compared with female. However, subgroup analysis stratified by sex showed no significant interaction between sex and PP levels for RHF suggesting that the association of increased risk for RHF with high PP levels was regardless of sex. Previous studies reported that high PP levels or ISH are typical in male than female in young adults (48). Spurious systolic hypertension or sympathetic overactivity observed in healthy young men are suggested as a mechanism for high PP levels (21, 22, 40, 49, 50). However, as noted in the previous studies, high PP levels in young men were a result of high arterial distensibility or hyperkinetic state and were considered as benign condition. It is unclear whether high PP levels in young men contribute to adverse effects on vascular physiology. However, as shown in the present study, the RHF associated with high PP levels may not be affected by sex differences. Further in-depth studies are needed to elucidate the sex-specific effects of PP on renal vascular physiology in young adults.

This study had several limitations. First, direct measurement of GFR was not performed in this study. The gold standard methods to assess GFR include direct measurement of renal clearance of endogenous or exogenous substances (51, 52). However, the KNHANES data was designed for the purpose of national health examination and direct measurement of renal clearance was not included. Second, the definition of RHF is not generally defined in children to young adults. Some of studies used cutoff criteria with directly measured GFR or eGFR to define RHF (3, 53). In this study, multivariable linear regression methods calculating RHF adjusted for logarithm-transformed age, sex, weight and height were used to reduce the possible confoundings. Further studies are needed to validate the definition of RHF in young adults. Third, the interpretation of subgroup analyses stratified by histories of diabetes or dyslipidemia and ISH or IDH are limited. The numbers of participants with diabetes or dyslipidemia and ISH or IDH were small as this study was consisted of relatively healthy young adults aged 19 to 39 years. Further study with large number of subjects is warranted to confirm the subgroup effects between PP and RHF in young adults. Finally, due to the observational nature of this study, the causal relationship between high PP and the risk of RHF cannot be demonstrated. Further longitudinally design studies should be performed to affirm the present study findings.

In conclusion, high PP is associated with an increased risk of RHF in young adults with no history of hypertension and with normal kidney function. In particular, obese young adults with elevated PP levels may show more increased risk of RHF. Considering that early identification and management of kidney injury starting from the young adults may prevent progression of kidney disease, assessment of PP and associated RHF may give benefit to early detect the potential risk of CKD development in young adults.

## Data availability statement

The datasets presented in this study can be found in online repository. The names of the repository and accession numbers can be found at: https://knhanes.kdca.go.kr/knhanes/sub03/sub03_01.do.

## Ethics statement

The studies involving human participants were reviewed and approved by Institutional Review Board (IRB) of the Centers for Disease Control and Prevention in Korea. The patients/participants provided their written informed consent to participate in this study. Written informed consent was obtained from the individual(s) for the publication of any potentially identifiable images or data included in this article.

## Author contributions

JHJ contributed to the research idea and study design. EJY was responsible for data acquisition. EJY, SHP, SYL, and DHO contributed to the data analysis/interpretation. EJY and DHO performed the statistical analysis. HYC and HCP were responsible for supervision or mentorship. JHJ was the guarantor. All authors contributed to the important intellectual content during manuscript drafting or revision and accepts accountability for the overall work by ensuring that questions pertaining to the accuracy or integrity of any portion of the work are appropriately investigated and resolved.

## Supplementary material

Supplementary Table 1 Baseline characteristics in male (*n* = 4,649).

Supplementary Table 2 Baseline characteristics in female (*n* = 5,716).

Supplementary Table 3 The regression coefficients for the risk of RHF.

Supplementary Table 4 Subgroup analysis stratified by sex, BMI, histories of diabetes or dyslipidemia, and ISH or IDH.
