# Supplementary material for: Pulse pressure and the risk of renal hyperfiltration in young adults: Results from Korea National Health and Nutrition Examination Survey (2010–2019)
Source: Front Med (Lausanne). 2022 Sep 13;9:911267. doi: 10.3389/fmed.2022.911267 (PMC9513024; doi:10.3389/fmed.2022.911267)
Supplement: Supplementary Table 1 — Baseline characteristics in male (n = 4,649). [file Data_Sheet_1.docx]

**Supplementary Material**

**Pulse Pressure and the Risk of Renal Hyperfiltration in Young Adults: Results from Korea National Health and Nutrition Examination Survey (2010-2019)**

Eunji Yang, M.D.^1^, Sang Ho Park, M.S.^2^, Seoyoung Lee, M.D.^1^, Donghwan Oh, M.D.^1^, Hoon Young Choi, M.D., Ph.D.^1,3^, Hyeong Cheon Park, M.D., Ph.D.^1,3^, and Jong Hyun Jhee, M.D., Ph.D.^1^

*^1^Division of Nephrology, Department of Internal Medicine, Gangnam Severance Hospital, Yonsei University College of medicine, Seoul, Republic of Korea*

*^2^Department of Internal Medicine, Gangnam Severance Hospital, Yonsei University College of medicine, Seoul, Republic of Korea*

*^3^ Severance Institute for Vascular and Metabolic Research, Yonsei University College of Medicine, Seoul, Republic of Korea*

**Corresponding Author:**

Jong Hyun Jhee, M.D., Ph.D.

Division of Nephrology, Department of Internal Medicine, Gangnam Severance Hospital, Yonsei University College of Medicine, 211 eonju-ro, Gangnam-gu, Seoul 06273, Republic of Korea

Phone: 82-2-2019-4368; Fax: 82-2-3463-3882; E-mail: [jjhlove77@yuhs.ac](mailto:jjhlove77@yuhs.ac)

**Content list:**

**Supplementary Table 1.** Baseline characteristics in male (n = 4,649)

**Supplementary Table 2.** Baseline characteristics in female (n = 5,716)

**Supplementary Table 3.** The regression coefficients for the risk of RHF

**Supplementary Table 4.** Subgroup analysis stratified by sex, BMI, histories of diabetes or dyslipidemia, and ISH or IDH

**Supplementary Table 1.** Baseline characteristics in male (n = 4,649)

|  | **Pulse pressure** | | | |
| --- | --- | --- | --- | --- |
| **Characteristics** | **T1**  **(n=1,475)** | **T2**  **(n=1,707)** | **T3**  **(n=1,467)** | ***P*** |
| Demographic data |  | | |  |
| Age, years | 31.1 ± 6.0 | 30.1 ± 6.0 | 27.9 ± 6.2 | <0.001 |
| BMI, kg/m^2^ | 23.8 ± 3.5 | 24.2 ± 3.6 | 25.0 ± 4.0 | <0.001 |
| Smoking status, n (%) | 947 (64.2%) | 1117 (65.4%) | 886 (60.4%) | 0.010 |
| Alcohol status, n (%) | 1439 (97.6%) | 1669 (97.8%) | 1427 (97.3%) | 0.661 |
| SBP, mmHg | 107.0 ± 7.0 | 113.4 ± 7.5 | 120.3 ± 8.4 | <0.001 |
| DBP, mmHg | 76.7 ± 6.6 | 75.5 ± 7.4 | 72.4 ± 8.3 | <0.001 |
| PP, mmHg | 30.3 ± 3.3 | 37.9 ± 1.9 | 47.9 ± 5.7 | <0.001 |
| Education, n (%) |  | | | 0.747 |
| Low | 24 (1.6%) | 29 (1.7%) | 29 (2.0%) |  |
| High | 1451 (98.4%) | 1678 (98.3%) | 1438 (98.0%) |  |
| Income, n (%) |  | | | 0.760 |
| Low | 737 (50.0%) | 848 (49.7%) | 714 (48.7%) |  |
| High | 738 (50.0%) | 859 (50.3%) | 753 (51.3%) |  |
| Comorbidities, n (%) |  | | |  |
| Diabetes | 9 (0.6%) | 10 (0.6%) | 11 (0.7%) | 0.830 |
| Dyslipidemia | 45 (3.1%) | 48 (2.8%) | 34 (2.3%) | 0.460 |
| Cardiovascular disease | 2 (0.1%) | 2 (0.1%) | 1 (0.1%) | 0.443 |
| Laboratory data |  | | | |
| eGFR, mL/min/1.73 m^2^ | 104.3 ± 12.6 | 106.2 ± 12.6 | 108.9 ± 13.0 | <0.001 |
| Proteinuria, n (%) | 202 (13.7%) | 244 (14.3%) | 223 (15.2%) | 0.503 |
| Hemoglobin, g/dL | 15.7 ± 1.0 | 15.6 ± 0.9 | 15.6 ± 1.0 | 0.216 |
| Fasting plasma glucose, g/dL | 92.9 ± 12.9 | 93.6 ± 17.8 | 93.1 ± 13.0 | 0.324 |
| HbA1c, % | 5.42 ± 0.46 | 5.42 ± 0.52 | 5.40 ± 0.48 | 0.494 |
| Total cholesterol, mg/dL | 188.3 ± 36.3 | 187.7 ± 33.4 | 183.4 ± 32.8 | <0.001 |
| LDL-C, mg/dL | 111.3 ± 34.4 | 110.7 ± 31.2 | 106.2 ± 31.0 | <0.001 |
| ***Note:*** Data are presented as mean ± SD and number (%).  ***Abbreviations:*** BMI, body mass index; SBP, systolic blood pressure; DBP, diastolic blood pressure; PP, pulse pressure; eGFR, estimated glomerular filtration rate; LDL-C, low density lipoprotein-cholesterol; SD, standard deviation | | | | |

**Supplementary Table 2.** Baseline characteristics in female (n = 5,716)

|  | **Pulse pressure** | | | |
| --- | --- | --- | --- | --- |
| **Characteristics** | **T1**  **(n=2,167)** | **T2**  **(n=1,339)** | **T3**  **(n=2,210)** | ***P*** |
| Demographic data |  | | |  |
| Age, years | 30.9 ± 5.9 | 30.7 ± 6.1 | 29.9 ± 6.3 | <0.001 |
| BMI, kg/m^2^ | 21.7 ± 3.3 | 22.0 ± 3.5 | 22.6 ± 3.9 | <0.001 |
| Smoking status, n (%) | 411 (19.0%) | 222 (16.6%) | 367 (16.6) | 0.073 |
| Alcohol status, n (%) | 73 (3.4%) | 50 (3.7%) | 103 (4.7%) | 0.081 |
| SBP, mmHg | 100.0 ± 7.0 | 104.2 ± 7.2 | 109.7 ± 8.7 | <0.001 |
| DBP, mmHg | 71.5 ± 6.7 | 69.8 ± 7.2 | 68.1 ± 8.4 | <0.001 |
| PP, mmHg | 28.5 ± 3.1 | 34.4 ± 1.1 | 41.6 ± 4.5 | <0.001 |
| Education, n (%) |  | | | 0.284 |
| Low | 42 (1.9%) | 35 (2.6%) | 57 (2.6%) |  |
| High | 2125 (98.1%) | 1304 (97.4%) | 2153 (97.4%) |  |
| Income, n (%) |  | | | 0.076 |
| Low | 1020 (47.1%) | 654 (48.8%) | 1116 (50.5%) |  |
| High | 1147 (52.9%) | 685 (51.2%) | 1094 (49.5%) |  |
| Comorbidities, n (%) |  | | |  |
| Diabetes | 17 (0.8%) | 6 (0.4%) | 13 (0.6%) | 0.450 |
| Dyslipidemia | 22 (1.0%) | 30 (2.2%) | 28 (1.3%) | 0.009 |
| Cardiovascular disease | - | - | - |  |
| Laboratory data |  | | | |
| eGFR, mL/min/1.73 m^2^ | 112.2 ± 11.4 | 113.3 ± 11.4 | 114.8 ± 11.3 | <0.001 |
| Proteinuria, n (%) | 337 (15.6%) | 151 (11.3%) | 321 (14.5%) | 0.002 |
| Hemoglobin, g/dL | 13.2 ± 1.0 | 13.1 ± 1.0 | 13.0 ± 1.2 | <0.001 |
| Fasting plasma glucose, g/dL | 89.8 ± 11.7 | 90.7 ± 17.4 | 90.7 ± 13.3 | 0.070 |
| HbA1c, % | 5.34 ± 0.47 | 5.36 ± 0.56 | 5.37 ± 0.46 | 0.086 |
| Total cholesterol, mg/dL | 182.8 ± 31.7 | 181.3 ± 32.1 | 180.5 ± 32.1 | 0.057 |
| LDL-C, mg/dL | 106.9 ± 27.1 | 105.3 ± 27.9 | 104.4 ± 27.6 | 0.008 |
| ***Note:*** Data are presented as mean ± SD and number (%).  ***Abbreviations:*** BMI, body mass index; SBP, systolic blood pressure; DBP, diastolic blood pressure; PP, pulse pressure; eGFR, estimated glomerular filtration rate; LDL-C, low density lipoprotein-cholesterol; SD, standard deviation | | | | |

**Supplemental table 3.** Regression coefficients for the risk of RHF

|  | Unadjusted | | | Fully adjusted | | |
| --- | --- | --- | --- | --- | --- | --- |
|  | **B** | **OR (95% CI)** | ***P*** | **B** | **OR (95% CI)** | ***P*** |
| Pulse pressure |  |  |  |  |  |  |
| Per 1.0 mmHg | 1.61 | 4.98 (3.61 – 6.86) | <0.001 | 0.85 | 2.36 (1.67 – 3.32) | <0.001 |
| T1 | (Reference) | | | | | |
| T2 | 0.50 | 1.65 (1.39 – 1.96) | <0.001 | 0.35 | 1.42 (1.19 – 1.69) | <0.001 |
| T3 | 0.72 | 2.05 (1.73 – 2.44) | <0.001 | 0.36 | 1.44 (1.20 - 1.73) | <0.001 |
| Age | 0.00 | 0.97 (0.99 – 1.01) | 0.424 | -0.01 | 0.99 (0.98 – 1.00) | 0.187 |
| Sex | -1.22 | 0.30 (0.26 – 0.34) | <0.001 | -2.04 | 0.13 (0.10 – 0.17) | <0.001 |
| BMI | -0.04 | 1.05 (1.03 – 1.06) | <0.001 | 0.00 | 1.00 (0.98 – 1.02) | 0.987 |
| Education | -0.67 | 0.51 (0.36 – 0.73) | <0.001 | -0.73 | 0.48 (0.33 – 0.70) | <0.001 |
| Income | -0.13 | 0.88 (0.77 – 1.00) | 0.055 | -0.10 | 0.91 (0.80 – 1.04) | 0.162 |
| Smoking | 0.59 | 1.81 (1.59 – 2.06) | <0.001 | 0.10 | 1.11 (0.95 – 1.29) | 0.198 |
| Alcohol | -0.07 | 0.94 (0.66 – 1.33) | 0.711 | -0.16 | 0.85 (0.59 – 1.23) | 0.393 |
| Diabetes | 0.89 | 2.44 (1.35 – 4.43) | 0.003 | 1.01 | 2.74 (1.46 – 5.14) | 0.002 |
| Hb | 0.15 | 1.17 (1.12 – 1.21) | <0.001 | -0.35 | 0.71 (0.66 – 0.75) | <0.001 |
| TC | - | 1.00 (1.00 – 1.003) | 0.188 | - | 1.00 (1.00 – 1.004) | 0.081 |
| PU | -0.13 | 0.87 (0.72 – 1.06) | 0.168 | -0.10 | 0.90 (0.74 – 1.10) | 0.302 |

***Abbreviation***: B, regression coefficient; OR, odds ratio; CI, confidence interval; BMI, body mass index; Hb, hemoglobin; TC, total cholesterol; PU, proteinuria

**Supplementary Table 4.** Subgroup analyses stratified by sex, BMI, histories of diabetes or dyslipidemia, and ISH or IDH

| Subgroup | | Prevalence RHF/total, n (%) | OR (95% CI)^*^ | *P* for interaction |
| --- | --- | --- | --- | --- |
| Sex | Female | 301/5,716 (5.3%) | 2.17 (1.15 – 4.10) | 0.59 |
|  | Male | 735/4,649 (15.8%) | 2.37 (1.57 – 3.58) |  |
| BMI | <25 kg/m^2^ | 686/7,599 (9.0%) | 1.89 (1.25 – 2.87) | 0.01 |
|  | ≥25 kg/m^2^ | 350/2,766 (12.7%) | 3.16 (1.74 – 5.73) |  |
| Dyslipidemia | Yes | 27/207 (13.0%) | 1.30 (0.95 – 1.78) | 0.65 |
|  | No | 1,009/10,158 (9.9%) | 1.09 (1.05 – 1.13) |  |
| Diabetes | Yes | 14/66 (21.2%) | 1.66 (0.87 – 3.16) | 0.24 |
|  | No | 1,022/10,299 (9.9%) | 1.09 (1.05 – 1.13) |  |
| ISH | Yes | 11/75 (14.7%) | 1.33 (0.45 – 3.92) | 0.30 |
|  | No | 1,025/10,290(10.0%) | 1.09 (1.06 – 1.13) |  |
| IDH | Yes | 217/1,774 (12.2%) | 2.31 (0.94 – 5.70) | 0.08 |
|  | No | 819/8,591 (9.5%) | 2.50 (1.70 – 3.68) |  |
| *Note*: ^*^ORs were calculated per 1.0 increase of log-transformed PP levels after adjustment for age, sex, BMI, income, education, alcohol and smoking status, history of diabetes, hemoglobin, total cholesterol, and proteinuria  *Abbreviation*: BMI, body mass index; RHF, renal hyperfiltration; OR, odds ratio; CI, confidence interval; ISH, isolated systolic hypertension; IDH, isolated diastolic hypertension | | | | |
